# LULC Classification and Topographic Correction of Landsat-7 ETM+ Imagery in the Yangjia River Watershed: the Influence of DEM Resolution

**DOI:** 10.3390/s90301980

**Published:** 2009-03-17

**Authors:** Yongnian Gao, Wanchang Zhang

**Affiliations:** 1 International Institute for Earth System Science (ESSI), Nanjing University. Nanjing 210093, P.R. China; 2 Key Laboratory of Regional Climate-Environment Research for Temperate East Asia, Institute of Atmospheric Physics, CAS, Beijing 100029, P.R. China

**Keywords:** Land Use/Land Cover (LULC), SCS Correction, Minnaert correction, DEM, Landsat-7 ETM+

## Abstract

DEM-based topographic corrections on Landsat-7 ETM+ imagery from rugged terrain, as an effective processing techniques to improve the accuracy of Land Use/Land Cover (LULC) classification as well as land surface parameter retrievals with remotely sensed data, has been frequently reported in the literature. However, few studies have investigated the exact effects of DEM with different resolutions on the correction of imagery. Taking the topographic corrections on the Landsat-7 ETM+ images acquired from the rugged terrain of the Yangjiahe river basin (P.R. China) as an example, the present work systematically investigates such issues by means of two commonly used topographic correction algorithms with the support of different spatial resolution DEMs. After the pre-processing procedures, i.e. atmospheric correction and geo-registration, were applied to the ETM+ images, two topographic correction algorithms, namely SCS correction and Minnaert correction, were applied to assess the effects of different spatial resolution DEMs obtained from two sources in the removal of topographic effects and LULC classifications. The results suggested that the topographic effects were tremendously reduced with these two algorithms under the support of different spatial resolution DEMs, and the performance of the topographic correction with the 1:50,000-topographic-map DEM was similar to that achieved using SRTM DEM. Moreover, when the same topographic correction algorithm was applied the accuracy of LULC classification after topographic correction based on 1:50,000-topographic-map DEM was similar as that based on SRTM DEM, which implies that the 90 m SRTM DEM can be used as an alternative for the topographic correction of ETM+ imagery when high resolution DEM is unavailable.

## 1. Introduction

LULC maps as necessary inputs for distributed eco-hydrological models are very essential for eco-hydrological process modeling, and LULC mapping with remotely sensed data by means of different classification algorithms has become a popular approach. However, serious topographic effects, i.e. the surface oriented towards the sun receives more radiance than that oriented away from the sun on the opposite slope [1,2], have been usually found in the remotely sensed imageries acquired from undulating mountainous or hilly lands, especially for the high spatial resolution images such as Landsat-7 ETM+. Topographic effects have been recognized as an important factor responsible for the existence of the same object appearing in different spectral response or visa verse. The negative effects of these phenomena on the accurate classification of LULC with remote sensing techniques have been amply discussed in the literature [3,4], and topographic correction is the common approach to reduce the topographic effects before further processing and analysis being applied for the ETM+ images from rugged terrain.

Various correction algorithms using digital elevation model (DEM) have been proposed to account for this problem as a preliminary step to the digital classification of LULC for specific sensors. Among these algorithms, cosine correction [5], C correction [5], b correction [6], two-stage normalization [7], SCS correction [3], SCS+C correction [8], Minnaert correction [9] and so on are world-wide utilized techniques. The most important issue for DEM-based topographic correction is DEM resolution and availability [10]. High-resolution DEM provides basic topographic information of the target area of the scene to facilitate specific algorithm to reduce the topographic effects of the scene. By calculating the slope and aspect of a surface, the sun-surface/canopy-sensor orientation model can be included in standard satellite image analyses [10]. Reeder [10] pointed out that the improvements in the availability of high-resolution DEM throughout the United States and globally suggested that the topographic correction methods would be gained widespread use in the remote sensing community. Conese *et al*. [11] thought that the resolution and accuracy of DEM would influence the performance of topographic correction, and this viewpoint has been widely accepted for most researchers, but different opinion existed on the issue of DEM resolutions [12–14]. Some of them claimed that the influence of DEM resolution on topographic correction should not be less than that of the image resolution, and even some of them stated that the influence of DEM resolution should be as four times strong as that of the image resolution. Civco [7] stated that better accuracy could be obtained in topographic corrections when the DEM used in calculating sun-surface-sensor orientation had the same or better resolution than the satellite image. These different recognitions on the influence of accuracy and resolution of DEM in topographic corrections mainly originated from the lack of systematic investigations on effects of different spatial resolution DEMs on topographic correction and LULC classification of remotely sensed images. High-accuracy and high-resolution DEM was usually expensive and difficult to obtain for researchers, which, in some extent, has restricted the development and application of topographic correction models in the past. Global 90 m high-resolution DEM data derived from the Shuttle Radar Topography Mission (SRTM) has become widely available in recent years, however, whether this data is sufficient in accuracy and spatial resolution for topographic corrections on Landsat TM/ETM+ images, yet remained as an unresolved issue in earlier published studies [10]. This study was thus aimed to address the effects of different spatial resolution DEM on performances of topographic correction and LULC classification of remotely sensed images by some well designed experiments.

In this study, the Yangjia river watershed, located on the south flank of Qiliang Mountain (P.R. China) was selected as a test area, the SRMT 90 m DEM and the 30 m DEM constructed from 1:50,000 topographic map were selected as two sources of different resolution DEMs to facilitate topographic correction with SCS and Minnaert correction algorithms on Landsat ETM+ image acquired in the test site, immediately after the topographic correction, atmospheric correction was applied to the image and the unsupervised classifications were thus done to derived LULC maps of the study area. This process makes it possible to investigate and compare the exact effects of the two resolution DEMs on the topographic corrections as well as on LULC classification of the Landsat ETM+ images in some detail.

## 2. Methodology

### 2.1. Study Area Description

**Figure 1.**
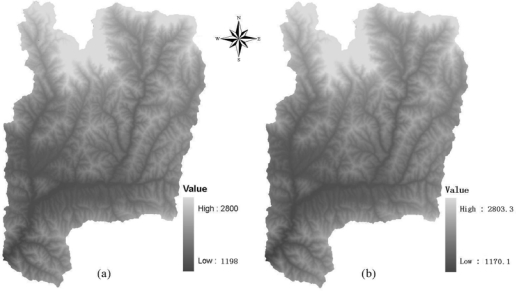
Different spatial resolution DEMs of the Yangjiahe river watershed, (a) represents the 30m DEM constructed with 1:50,000 topographic map; (b) represents the 30m DEM re-sampled with 90 m SRTM DEM.

Located on the south flank of Qinling Mountain in Taibai and Feng counties, Shanxi Province (106°59′33″−107°16′21″ E, 33°53′44″ −34°10′07″ N), P.R. China, the Yangjia river watershed is just one of three tributary catchment of the Bao river basin. Three tributaries, i.e. the Yangjia river, Huangniu river and Shigou river systems constitute the Bao river basin, among which the drainage area of the Yangjia river watershed occupies about 430 km^2^. Selection of this watershed as study area was mainly for two reasons, i.e.: 1) Undulating terrain characterizes the topography of the study site, as shown in Figure 1, where the field expeditions have been conducted in the past for LULC classifications, which was quite suitable for the purpose of the study; 2) Elevation ranges from about 1,170 to 2,800 m with an average of around 1,800 m, where the 30 m DEM constructed from 1:50,000 topographic map was made available by the previous researchers. This area belongs to typical temperate mountainous climate type with an annual mean temperature of approximately 11.4 °C and mean annual rainfall of approximately 613 mm. The LULC in the area mainly consists of forest, grass, bush etc., and the vegetation coverage ratio is rather high.

### 2.2. Data and Processing

The technical flow of this research includes six steps, i.e. generation of DEM, atmospheric correction, mask, topographic correction, assessment of correction performance and LULC classification.

#### 2.2.1. Generation of DEM

DEM data utilized in this study come from two sources. One was generated from digitized contour lines from 1:50,000 scale topographic map (20 m interval between each contour lines) and sampled to 30 m in spatial resolution to keep identical space resolution to that of Landsat-7 ETM+ image. Another was SRTM 90 m DEM which was downloaded, in a standardized GeoTIFF format, from ftp://ftp.glcf.umiacs.umd.edu and re-sampled to 30 m in spatial resolution using cubic convolution interpolation. The boundary map of the Yangjia river basin was generated from the DEM with ARCGIS software, and then the DEMs of Yangjia river basin were musked out with the watershed boundary (See Figure 1). Table 1 lists the summary statistics of this two different spatial resolution DEMs of the Yangjia river basin. It can be found that the DEMs from two different sources have similar mean, median, standard deviation, minimum and maximum values.

#### 2.2.2. Atmospheric Correction

The remotely sensed data used in this study was Landsat-7 ETM+ image (path 128/row 36) at spatial resolution 28.5 m × 28.5 m acquired on May 19, 2000. The solar zenith angle is 65.30°, the solar azimuth angle 120.98°. The image was obtained in a standardized orthorectified GeoTIFF format downloaded from ftp://jkjdl.jaflal.edu.com.

**Figure 2.**
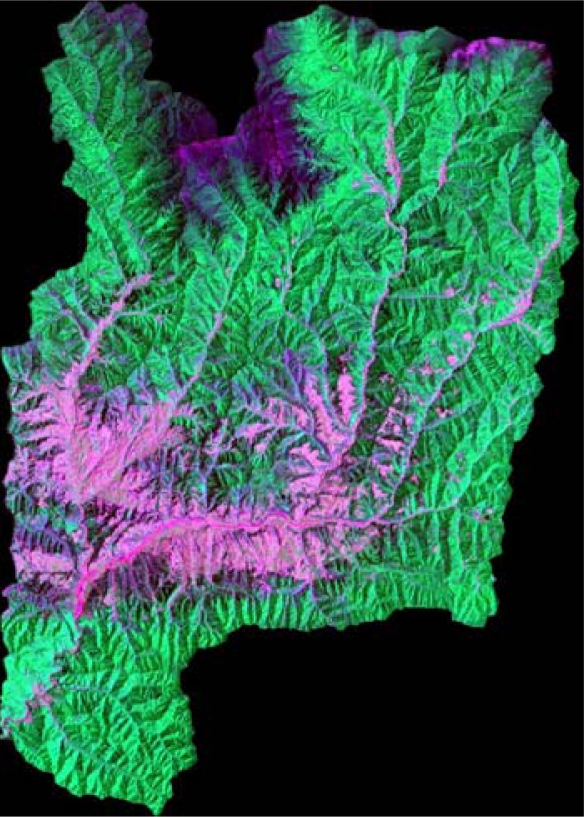
Color composite of RGB-543 Landsat-7 ETM+ image of the study watershed before topographic correction.

After being re-sampled to 30 m spatial resolution, the image was re-sized to fit the test area of the study site to be identical to that of DEM (Figure 1), and then the pixel *DN* values of image were converted to at-satellite radiance for each band following Mausel *et al.* [15]. The image-based atmospheric correction method, i.e. COST method originally proposed by Chavez [16] and was modified by Zhang *et al*. [17], was used to remove the atmospheric effects from the image. After topographically, atmospherically corrected, the Landsat-7 ETM+ image only encompassing the Yangjia river watershed (as shown in Figure 2) was finally extracted by the basin boundary map with ARCGIS software.

#### 2.2.3. Topographic Correction

Two widely used topographic correction methods, namely the SCS correction and Minnaert correction algorithms, were used to remove the topographic effects on the Yangjia river watershed image.

SCS correction was proposed by Gu *et al*. [3] based on sun-canopy-senor geometry, and it can be expressed as:

(1) $$L_{m}=L\cdot \left(\frac{\text{cos}\;\theta \cdot \text{cos}\;\alpha }{\text{cos}\;i}\right)$$

where *L_m_* is the normalized radiance, *L* is the uncorrected radiance, *θ* is the solar zenith angle, *i* is the incident angle, *α* is the slope of the surface. The SCS correction algorithms were developed under the assumption of Lambertian surface which implies terrain reflects irradiance equally in all directions. The assumption is not real in natural surfaces since most land covers are undulating with non-Lambertian characteristics [8,10,18]. Unlike Lambertian SCS correction, Minnaert correction, which introduces a parameter *k* to quantify the reflectance response over the natural terrain, is a non-Lambertian correction algorithm as following [9,12,19]:

(2) $$L_{m}=L\left(\text{cos}\;\alpha \right)/\left({\text{cos}}^{k}\;i\;{\text{cos}}^{k}\;\alpha \right)$$

where *k* is Minnaert constant which was proposed by Minnaert in 1941 [20] and mainly used for photometric analysis of lunar surface [21]. In Minnaert correction algorithm, the Minnaert constant *k* is mainly used to adjust corrections and its value ranges from 0 to 1 [9,22]. The value of *k* for each band can be calculated and obtained as follows. Firstly equation (2) can be transformed as:

(3) $$L\;\text{cos}\;\alpha =L_{m}\;{\text{cos}}^{k}\;i\;{\text{cos}}^{k}\;\alpha$$

(4) $$\text{ln}\left(L\;\text{cos}\;\alpha \right)=\text{ln}\;L_{m}+k\;\text{ln}\left(\text{cos}\;i\;\text{cos}\;\alpha \right)$$

let *x* = ln(cos *i* cos *α*), *y* = ln(*L* cos *α*), *c* = ln *L_m_*, the equation (4) can be expressed as *y* = *kx* + *c*, then the value of *k* can be estimated by regression method [9,12,19]. Table 2 presents the values of *k* parameter for Minnaert correction based on different spatial resolution DEMs of the Yangjia river basin.

#### 2.2.4. Assessment of Correction Performance

Visual comparison and statistical analysis were adopted to evaluate the performance of the corrections based on the two different spatial resolution DEMs. Scatter-plot and fitting line of reflectance *ρ* versus cos*i*, the slope *m* and correlation coefficient *r* of the linear regression equation, Relative Correction Extent (RCE), Dispersion Indices (DI) were used to quantitatively compare the effects of different spatial resolution DEMs on topographic correction.

The RCE for each band of the Landsat-7 ETM+ image before and after the correction can be expressed as:

(5) $$R=\frac{V_{m}-V}{V}\times 100\%$$

where *R* represents RCE, *V* and *V_m_* represent the absolute value of slope or correlation coefficient of the linear regression equation between reflectance *ρ* and cos*i* before and after topographic correction respectively.

The DI is calculated by:

(6) $$\mathrm{DI}=\frac{\mathrm{SD}}{M}\times 100\%$$

where *DI* is the dispersion index, *M* and *SD* represent the mean value and standard deviation of target area for each band of the Landsat-7 ETM+ image before and after the correction respectively.

#### 2.2.5. LULC Classification

A geographical database including spatial data and ground truth has been compiled for the Yangjia river watershed. It contains 1:200,000-scale forest distribution map of the Baoji city, 1 km spatial resolution land cover classification map of China for 2000 provided by Data Center for Resources and Environmental Sciences, Chinese Academy of Sciences (RESDC) and land cover information from field survey. The database was used to comprehensively perform the LULC classification and accuracy assessment.

The image was classified using a six category classification scheme, and the six classification categories consisted of forest land, cultivated land, suitable land for forest, rangeland, brush land and bare land. Spectral signatures were created for these six LULC classes of interest using the training data provided from the geographical database, and the classifications were performed using the Gaussian maximum likelihood (GML) classifier. The reason for adopting GML classifier for classification was mainly due to its outstanding performance in considering means, variances and co-variances of training site statistics which favors this classifier, used worldwide and relatively convenient to implement and robust for classification [23]. In this study, four classification schemes were designed as follows:

Scheme 1: GML classifier was applied to the image after SCS correction based on the 90 m SRTM DEM for LULC classification;
Scheme 2: GML classifier was applied to the image after SCS correction based on the 30 m DEM constructed from 1:50,000 scale topographic map for LULC classification;
Scheme 3: GML classifier was applied to the image after Minnaert correction based on the 90 m SRTM DEM for LULC classification;
Scheme 4: GML classifier was applied to the image after Minnaert correction based on the 30 m DEM constructed from 1:50,000 scale topographic map for LULC classification;

Accuracy assessment was performed on the classified images consisting of the six categories under these four classification schemes to test and validate the methodology. For each classified image, a confusion matrix was developed, and overall accuracy and Kappa statistics, which assess overall classification accuracy by incorporating individual errors of omission and commission [24], has been recommended as a suitable accuracy measure in thematic classification for representing the whole confusion matrix to evaluate the agreement between the classification results and the ground truth data [23].

## 3. Results

### 3.1. Effects of DEM Resolution on Topographic Correction

**Figure 3.**
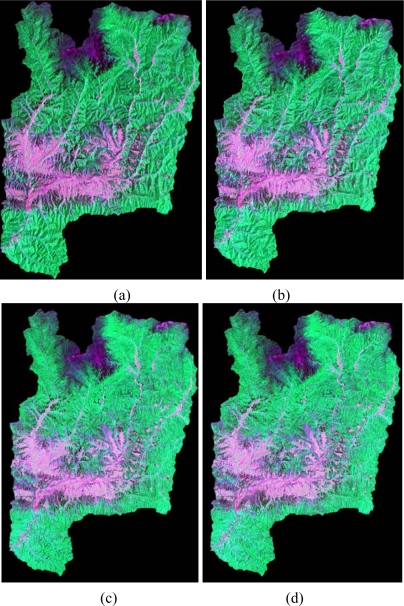
Comparison of color composite of RGB-543 Landsat-7 ETM+ image derived from two topographic correction methods being applied: (a) and (b) shows the image after SCS correction based on SRTM DEM and the DEM constructed from 1:50,000 scale topographic map respectively; (c) and (d) presents the image after Minnaert correction based on SRTM DEM and the DEM constructed from 1:50,000 scale topographic map respectively.

Figure 2 shows the false color composite image (ETM+ 5-4-3) before topographic correction, from which we can find that serious topographic effects appears on the image: the radiance in shaded areas show less than in sunny areas. Figures 3 (a)–(d) show the false color composite images (ETM+ 5-4-3) of the Yangjia river watershed after SCS and Minnaert correction under the support of different resolution DEMs, respectively. Figures 3(a) and (c) are based on SRTM DEM and Figures 3 (b) and (d) are based on DEM constructed from 1:50,000 scale topographic map.

In order to evaluate the performance of the topographic correction, statistical analyses were applied on the topographically corrected images. According to Reeder [10], successful topographic correction should remove or greatly reduce significant correlation of surface radiance with topographic variables, especially direct irradiance. We firstly depicted the scatter plots of reflectance *ρ* versus cos*i*, then the fitting lines were regressed linearly, and the slope *m* and correlation coefficient *r* of the linear regression equation were calculated and listed in Table 3.

Strong correlations can be found between the surface reflectance *ρ* and topographic variable cos*i* over the rugged terrain before correction. However, after topographic corrections with different resolution DEMs, both the slope *m* and correlation coefficient *r* were greatly reduced. It was noted that some of the slope *m* and correlation coefficients *r* are negative after SCS correction based on the two different DEMs. It indicates that the SCS algorithm has overcorrected the image. For the Minnaert correction, the *m* and *r* reduced dramatically compared with those before correction and they all are positive, which implies that none overcorrection existed for the Minnaert corrected images.

**Figure 4.**
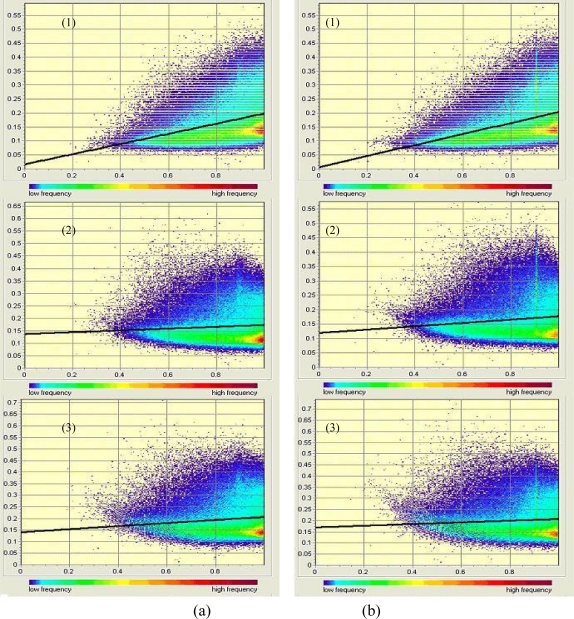
S scatter plots and the linear regression fitting lines of reflectance ρ versus cosi for ETM+ band 1 before and after correction: (a) based on 90 m SRTM DEM; (b) based on DEM constructed from 1:50,000 scale topographic map; (1) before topographic correction; (2) SCS correction; (3) Minnaert correction.

As an example, Figure 4 exhibits scatter plots and the linear regression fitting lines of reflectance *ρ* versus cos*i* for ETM+ band 1 before and after correction. It can be found that both the scatter plots and linear fitting lines after correction show almost horizontal distribution, which indicates that both the SCS and the Minnaert corrections yielded satisfactory results. Either based on the SRTM DEM or based the DEM constructed from 1:50,000 scale topographic map, both the SCS and the Minnaert correction model performed excellently in removing significant correlation between reflectance *ρ* and cos*i* for each band of Landsat-7 ETM+ image of the study site. From this study, we can find that based on these two different resolution DEMs with the same topographic correction method, the similar good performance of topographic correction can be obtained, which is consistent with the visual analysis concluded.

Following equation (6), the relative correction extent for each band of the Landsat-7 ETM+ image before and after the correction was calculated and listed in Table 4. It can be found that the absolute values for each band of the image after Minnaert correction based on the DEM constructed from 1:50,000 topographic map are less than those based on the SRTM DEM. It indicates that the Minnaert correction based on the DEM constructed from 1:50,000 topographic map performed more excellently than that based on the SRTM DEM in removing significant correlation between reflectance *ρ* and topographic variable cos*i* for each band of Landsat-7 ETM+ image. However, for SCS correction based on SRTM DEM, the absolute values for each band of the image, except bands 2 and 7, are bigger than that based on the DEM constructed from1:50,000 scale topographic map in various extent, which suggests that the SCS correction based on the SRTM DEM performed more excellently than that based on the DEM constructed from 1:50,000 topographic map in removing significant correlation between reflectance *ρ* and topographic variable cos*i* for each band of the Landsat-7 ETM+ image.

In order to further examine the accuracy of the topographic corrections statistically, the DI representing the quotient of standard deviations and mean of the study site for each band of the Landsat-7 ETM+ image before and after the corrections were calculated and listed in Table 5 for investigating spatial dispersion of spectral response of images before and after correction based on different DEM with different models.

From Table 5, we can see that the DI for each band of the images before topographic correction is relatively larger than that after topographic correction due to the serious topographic effects, except bands 3, 7 corrected by SCS model based on the SRTM DEM and band 3 corrected by SCS model based on the DEM constructed by 1:50,000 scale topographic map, which implies that based on the two different DEMs both SCS and Minnaert correction models are capable of removing topographic effect while improving overall quality of the image.

It was worthwhile to note that the DI values increase in order as the image after correction based on the DEM constructed from 1:50,000 scale topographic map, after correction based on the SRTM DEM and before correction with the same model, respectively. This implies that based on the DEM constructed from 1:50,000 scale topographic map the same correction model performed better than that based on the SRTM DEM.

### 3.2. Resolution Effects on Results of LULC Classification

**Figure 5.**
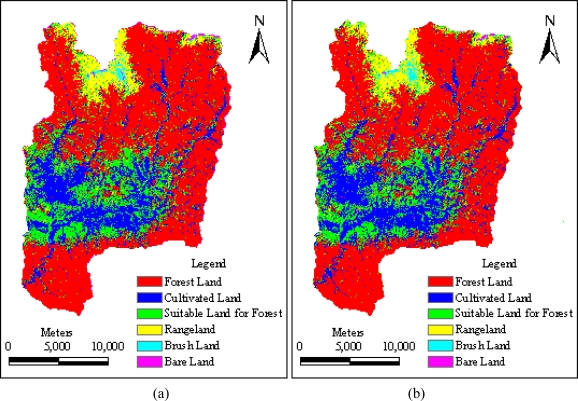

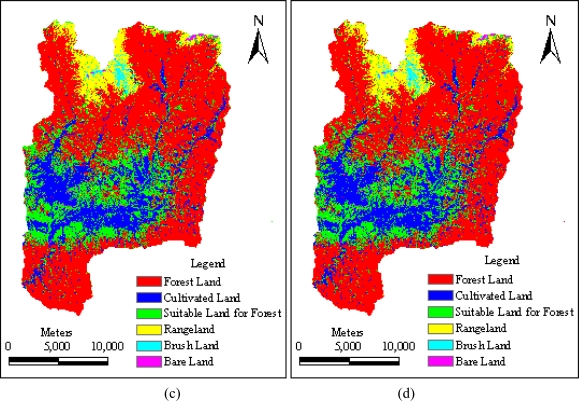
Image map of LULC Classification after topographic correction for the Yangjiahe river watershed: (a) and (b) illustrates the classification result after SCS correction based on SRTM DEM and the DEM constructed from 1:50,000 scale topographic map respectively; (c) and (d) exhibits the classification result after Minnaert correction based on SRTM DEM and the DEM constructed from 1:50,000 scale topographic map respectively.

In order to analyze the difference among the LULC classification results obtained from different correction methods and DEM resolutions, the classified images based only on spectral bands for the four different schemes were compared. Figure 5 presents the classification results obtained with those previously described four schemes. Visual comparison suggested that the four schemes successfully yielded quite similar classification results as shown in Figure 5.

Statistical method was adopted to further compare the classification performance of the four schemes. Overall classification accuracy and Kappa value that are widely used in accuracy assessment of LULC classification were computed and listed in Table 6. Closed overall classification accuracies and Kappa values derived from the four different classification schemes demonstrated that quantitative assessments agree with the visual assessments on classification accuracies of the image. Among four classification results, classification accuracies were found the lowest for the image performed by scheme 1 and highest for the image performed by scheme 3.

From Table 6, it also can be found that scheme 2 had a better classification performance with an overall accuracy of only 1.09% and Kappa value of only 0.01 higher than that of scheme 1, which implies that based on the DEM constructed from 1:50,000 scale topographic map the SCS correction can only improve the classification result slightly compared with that based on the SRTM DEM. However, it was beyond our expectation that scheme 4 had a worse classification performance with an overall accuracy of only 0.01% lower than that of scheme 3. It indicates that the classified image after Minnaert correction based on the SRTM DEM has better classification performance than that based on the DEM constructed from 1:50,000 scale topographic map.

## 4. Discussion and Conclusions

In this paper how the DEM resolution affects the performance of topographic correction and LULC classification accuracy on the processing of Landsat-7 ETM+ were systematically investigated for a case study on the Yangjia river watershed, Shanxi Province, P.R. China. The scatter plots and fitting lines of reflectance *ρ* versus cos*i*, the slope and correlation coefficients of the linear regression equation, relative correction extents, Dispersion Indices (DI), and the overall accuracies and Kappa values of the LULC classification obtained at different DEM resolutions were analyzed and compared. Visual comparison and quantitative statistic analyses on the topographically corrected and GLM classifier classified images derived from the different resolution DEMs being utilized were discussed in detail for classify the issue.

Some of the major findings from the experimental results can be summarized as follows:

1. Based on either the 90 m SRTM DEM or the 30 m DEM constructed from 1:50,000 scale topographic map, both SCS and Minnaert correction are able to successfully remove the topographic effects of the Landsat-7 ETM+ image in the Yangjia river watershed. And similar correction performances were obtained with the same topographic correction method being used under the support of either of these two different resolution DEMs.
2. The classified images after the same correction based on the two different DEMs give similar results. The overall accuracy and Kappa values of LULC classification are similar after SCS or Minnaert topographic corrections based on the above mentioned different spatial resolution DEMs.

In many cases, the high resolution DEM is not available for different reasons in many developing countries and other districts, which restricts the application of topographic corrections. However, the SRTM 90 m DEM for the entire world, which can be easily accessed, freely downloaded for public, will help to break this limitation. According to major findings in this study, we can make the SRTM 90 m DEM as an alternative for the topographic correction of Lanfsat-7 ETM+ images when lack of the high resolution DEM.

## Figures and Tables

**Table 1.** Statistic list of different spatial resolution DEMs of the Yangjia river watershed (m).

**Source of DEM**	**Mean**	**Median**	**Std. Deviation**	**Minimum**	**Maximum**

1:50,000 topographic map	1,808.588	1,773.7	313.495	1,198.0	2,800.0
SRTM	1,802.857	1,769.8	312.238	1,170.1	2,803.3

**Table 2.** Values of *k* parameter for Minnaert correction based on different spatial resolution DEMs.

**Source of DEM**	**Band 1**	**Band 2**	**Band 3**	**Band 4**	**Band 5**	**Band 7**

1:50,000 topographic map	0.940372	0.737627	0.459150	0.928376	0.781469	0.679558
SRTM	0.897112	0.708049	0.451846	0.897700	0.759341	0.650731

**Table 3.** Slope *m* and correlation coefficient *r* of regression model between ETM+ band 1–5, 7 reflectance and cos*i*.

**Source of DEM**	**Model**	**Statistics**	**Band 1**	**Band 2**	**Band 3**	**Band 4**	**Band 5**	**Band 7**

SRTM	Before correction	Slope *m*	0.18	0.26	0.17	0.11	0.08	0.05
		*r*	0.37	0.51	0.38	0.30	0.38	0.30

	SCS	Slope *m*	0.04	−0.02	−0.13	0.03	0.00	−0.01
		*r*	0.08	−0.04	−0.28	0.07	0.00	−0.06

	Minnaert	Slope *m*	0.07	0.09	0.07	0.04	0.02	0.02
		*r*	0.13	0.19	0.17	0.10	0.12	0.09

1:50000 topographic map	Before correction	Slope *m*	0.20	0.27	0.17	0.12	0.08	0.05
		*r*	0.43	0.57	0.41	0.34	0.42	0.34

	SCS	Slope *m*	0.06	0.00	−0.14	0.03	0.01	−0.01
		*r*	0.13	0.00	−0.31	0.10	0.03	−0.04

	Minnaert	Slope *m*	0.04	0.05	0.04	0.02	0.01	0.01
		*r*	0.08	0.11	0.11	0.06	0.06	0.06

**Table 4.** Relative correction extent for slope *m* and correlation coefficient *r* (unit: %).

**Source of DEM**	**Model**	**Statistics**	**Band 1**	**Band 2**	**Band 3**	**Band 4**	**Band 5**	**Band 7**

SRTM	SCS	Slope *m*	−80.10	−91.55	−21.70	−76.56	−99.28	−79.46
		*r*	−79.75	−91.42	−26.18	−76.29	−99.28	−80.00

	Minnaert	Slope *m*	−63.76	−64.68	−58.76	−64.68	−68.76	−67.34
		*r*	−65.53	−63.10	−56.59	−67.18	−69.61	−68.69

1:50,000 topographic map	SCS	Slope *m*	−70.59	−99.95	−19.51	−70.96	−93.93	−88.30
		*r*	−68.99	−99.94	−23.37	−70.17	−93.76	−88.36

	Minnaert	Slope *m*	−80.80	−81.62	−74.46	−80.28	−84.26	−82.27
		*r*	−81.99	−80.88	−73.12	−81.96	−84.82	−83.11

**Table 5.** Quotient comparison of SD and Mean for each band of the Landsat-7 ETM+ image before and after topographic correction (unit: %).

**Source of DEM**	**Model**	**Band 1**	**Band 2**	**Band 3**	**Band 4**	**Band 5**	**Band 7**

None	Before correction	42.41	23.47	21.54	54.44	34.12	37.29

SRTM	SCS	41.51	23.31	22.86	54.44	34.12	38.33
	Minnaert	38.17	19.76	19.75	50.48	30.21	35.38

1:50,000 topographic map	SCS	40.25	21.21	22.33	53.33	32.94	36.67
	Minnaert	37.50	19.70	17.38	50.00	30.61	34.85

**Table 6.** Comparison of the accuracy assessments of LULC classification after different topographic corrections based on different spatial resolution DEMs.

Classification schemes	Source of DEM	Model	Overall accuracy (%)	Kappa value
Scheme 1	SRTM	SCS	88.09	0.83
Scheme 3		Minnaert	89.68	0.85
Scheme 2	1:50000 topographic map	SCS	89.18	0.84
Scheme 4		Minnaert	89.67	0.85
